# The Role of Astrocytes in Synaptic Dysfunction and Memory Deficits in Alzheimer’s Disease

**DOI:** 10.3390/biom15070910

**Published:** 2025-06-20

**Authors:** Cristina A. Muñoz de León-López, Irene Navarro-Lobato, Zafar U. Khan

**Affiliations:** 1 Laboratory of Neurobiology, CIMES, University of Malaga, Campus Teatinos s/n, 29010 Malaga, Spain; 2 Department of Medicine, Faculty of Medicine, University of Malaga, Campus Teatinos s/n, 29010 Malaga, Spain; 3 CIBERNED, Institute of Health Carlos III, 28029 Madrid, Spain

**Keywords:** A1 phenotype, pro-inflammatory, ROS, gliotransmitters, synaptic plasticity, LTP, LTD, spike-time-dependent plasticity, homeostatic synaptic plasticity, synaptic remodeling, synaptogenesis, synaptic pruning, recognition memory, spatial memory, fear memory, working memory, treatment

## Abstract

Astrocytes are the most abundant glial cells in the brain. They play critical roles in synapse formation and function, neurotransmitter release and uptake, the production of trophic factors, and energy supply for neuronal survival. In addition to producing proteases for amyloid-β degradation, astrocytes express various receptors, transporters, gliotransmitters, and other molecules that enable them to sense and respond to external signals. They are also implicated in amyloid-β clearance. In Alzheimer’s disease, excessive accumulation of amyloid-β induces the polarization of astrocytes into the A1 phenotype, promoting the release of inflammatory cytokines and mitochondrial reactive oxygen species, leading to alterations in astrocytic functions. Under such conditions, gliotransmitter release, glutamate neurotransmission, AMPA receptor trafficking, and both Hebbian and non-Hebbian forms of synaptic plasticity—biological activities essential for synaptic functions—are compromised. Moreover, astrocytes are essential for learning, memory, and synaptic plasticity, and alterations in their function are associated with memory deficits in Alzheimer’s disease. This review provides an overview of the current understanding of the defects in astrocytes that lead to altered synaptic functions, neuronal structural plasticity, and memory deficits in Alzheimer’s disease.

## 1. Introduction

**Figure 1:**
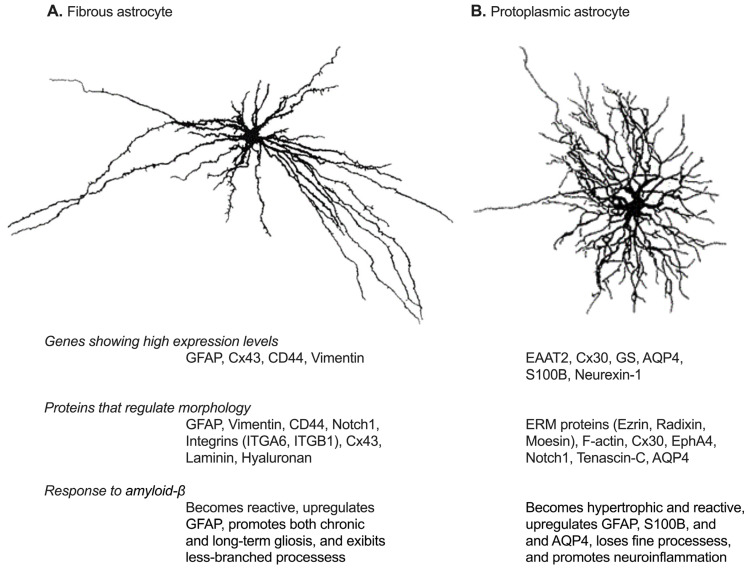
Astrocyte subtypes in the brain and their characteristics. (**A**) depicts fibrous astrocytes found in the white matter, characterized by long, straight, non-branched processes. In contrast, (**B**) represents protoplasmic astrocytes, which have a bushy or spongiform appearance with approximately 5–10 large primary processes radiating from the soma. The characteristics of each subtypes are described below their images. GFAP, glial fibrillary acidic protein; Cx43, connexin 43; CD44, cluster of differentiation 44; EAAT2, excitatory amino acid transporter 2; Connexin 30; GS, glutamine synthetase; AQP4, aquaporin 4; ITGA6, integrin alpha 6; ITGB1, integrin beta 1; F-actin, filamentous actin; EphA4, ephrin type-A receptor 4.

The brain consists of two main types of cells: neurons and glial cells. Neurons are excitable cells responsible for transmitting and processing information. They include excitatory pyramidal neurons and inhibitory GABAergic interneurons. In contrast, glial cells provide structural and functional support to neurons. The major types of glial cells include astrocytes, which support synapses, neurotransmitter recycling, and the blood-brain barrier; oligodendrocytes, which produce myelin for axonal insulation; and microglia, which act as immune cells by clearing debris and regulating inflammation. Among glial cells, astrocytes are the most abundant and are distributed ubiquitously throughout the brain [1]. Diverse astrocyte populations have been described in the brain and are classified based on morphological features [2,3]. Fibrous astrocytes are primarily localized in the white matter, while protoplasmic astrocytes are found in the gray matter (Figure 1). Both types of astrocytes Furthermore, astrocytes can also be classified based on distinct functional characteristics, such as synapse association [4,5], membrane properties, and Ca^2+^ signaling [5]. Astrocytes express a variety of receptors, transporters, and other molecules that enable them to sense and respond to external signals. While Ca^2+^ elevation is the primary response mechanism, astrocytes also integrate signals through potassium channels, cAMP and IP_3_ signaling, reactive oxygen species (ROS) and redox signaling, as well as cytokine and immune pathways [6]. They secrete various molecules, including γ-aminobutyric acid (GABA), adenosine triphosphate (ATP), D-serine, L-lactate, and brain-derived neurotrophic factor (BDNF), to regulate brain homeostasis, synaptic function, and cognitive processes such as emotion and memory [7,8].

Astrocytes play a critical role in synapse formation and function, neurotransmitter release and uptake, the production of trophic factors, and energy supply for neuronal survival [2,9,10]. Recent studies have shown that dysregulation of astrocyte function is linked to the initiation and progression of Alzheimer’s disease (AD) [11,12] as well as neuronal cell death in neurodegenerative diseases [13,14,15]. AD is a progressive neurodegenerative disease characterized by memory loss and cognitive dysfunction [16,17,18], with neuronal cell death as a prominent pathological feature [19,20]. Extracellular signals drive astrocyte polarization and their adaptation to different reactive states, often classified as the A1 phenotype, which is pro-inflammatory and neurotoxic, and the A2 phenotype, which is anti-inflammatory and neuroprotective [21,22]. In various pathological conditions, astrocytes undergo morphological, molecular, and functional alterations, transforming into reactive astrocytes [23,24] and contributing to neurodegeneration [25].

## 2. Reactive Astrogliosis in Alzheimer’s Disease

**Figure 2:**
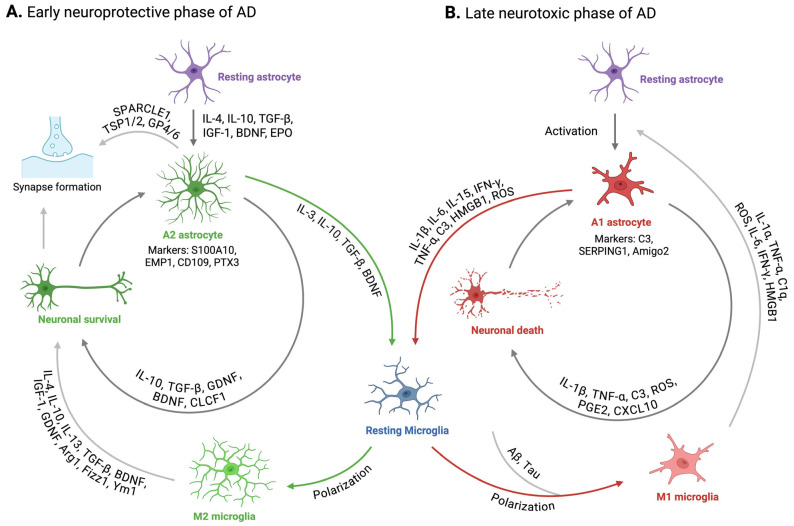
Intercommunication between astrocytes and microglia in AD. (**A**) illustrates the neuroprotective cycle during normal brain function and the early stages of AD. In response to extracellular signals or upon contact with amyloid-β and tau, astrocytes become activated and adopt an A2 reactive state. These A2 astrocytes release cytokines and growth factors that promote the polarization of microglia to the M2 phenotype. This polarization enhances the capacity of microglia to phagocytose amyloid-β and tau aggregates and supports neuronal survival and synaptic health. (**B**) depicts the dysfunctional cycle characteristic of the neurotoxic phase of AD. When microglia are unable to effectively clear excessive amyloid-β and tau deposits, they polarize toward the M1 phenotype, releasing proinflammatory cytokines and other factors that activate astrocytes and induce their transformation into the A1 phenotype. A1 astrocytes, in turn, release additional cytokines and mediators that further drive microglial activation and M1 polarization, establishing a positive feedback loop that contributes to neurotoxicity and neuronal cell death. IL-4, interleukin 4; IL-10, interleukin 10; IGF-1, insulin-like growth factor 1; BDNF, brain-derived neurotrophic factor; TGF-β, transforming growth factor beta; EPO, erythropoietin; SPARCLE1, SPARC-like 1, also known as hevin; TSP1/2, thrombospondin 1 and thrombospondin 2; GP4/6, glycoprotein 4 and glycoprotein 6; GDNF, glial cell line-derived neurotrophic factor; S100A10, S100 calcium binding protein A10; EMP1, epithelial membrane protein 1; CD109, cluster of differentiation 109; PTX3, pentraxin 3; IL-13, interleukin 13; Arg1, arginase 1; Fizz1, found in inflammatory zone 1; Ym1 also known as Chil3, chitinase-like protein 3; IL-1α, interleukin 1 alpha; TNF-α, tumor necrosis factor alpha; C1q, complement component 1q; ROS, reactive oxygen species; IL-6, interleukin 6; IFN-γ, interferon gamma; HMGB1, high mobility group box 1; IL-1β, interleukin 1 beta; C3, complement component 3; PGE2, prostaglandin E2; CXCL10, C-X-C motif chemokine ligand 10; IL-15, interleukin 15; Aβ, amyloid-β; SERPING1, serpin family G member 1; Amigo2, adhesion molecule with Ig like domain 2.

Reactive astrogliosis is a hallmark of AD. Morphological studies in post-mortem AD patient brains and mouse model of AD (AD mice) have shown a close interaction between astrocytes and amyloid-β [26]. Upon contact with amyloid-β, astrocytes become reactive, exhibiting morphological hypertrophy characterized by thicker processes and increased expression of intermediate filament proteins such as glial fibrillary acidic protein (GFAP), vimentin, nestin, and synemin [27,28]. This reactive astrogliosis has been shown to be mediated by intracellular Ca^2+^ signaling, as blocking Ca^2+^ release from the endoplasmic reticulum suppresses astrocytic reactivity [29]. Additionally, astrocytes not only express amyloid-β-degrading proteases that cleave amyloid-β into smaller fragments [30] but also play a crucial role in the glymphatic system, which is implicated in amyloid-β clearance [31,32]. The finding that amyloid-β accumulation increases in AD mice following the inhibition of astrogliosis [33] further suggests that this type of reactive astrogliosis may have beneficial roles in AD [15]. However, in the later stages of AD, excessive accumulation of amyloid-β induces the polarization of A2 astrocytes into the A1 phenotype, and the pro-inflammatory cytokines and ROS released by A1 astrocytes further worsen AD pathology by creating a positive feedback loop with microglia that enhances neuroinflammation diseases [14,34] and exacerbates amyloid-β accumulation [35,36] (Figure 2).

It has been shown that when aggregated tau, another protein implicated in AD pathology, comes into contact with astrocytes, it induces reactive astrogliosis, leading to their transformation into the A1 phenotype and the upregulation of GFAP and vimentin [37]. Moreover, tau can also accumulate within astrocytes, and these astrocytic tau aggregates alter intracellular Ca^2+^ signaling, disrupt the release of gliotransmitters, and cause neuronal dysfunction and memory deficits [38,39].

## 3. Dysregulation in Microglia-Astrocyte Interaction in Alzheimer’s Disease

The interplay between microglia and astrocytes is central to the pathogenesis and progression of AD. These glial cells work synchronously and complementarily to regulate amyloid-β deposition, tau aggregation, and neuroinflammation [40,41,42]. It has been shown that microglia, similar to astrocytes, cluster around amyloid plaques in AD mouse models as well as in patients [42,43] and are activated upon contact with amyloid-β [44]. In the early neuroprotective stage of AD, the accumulation of amyloid-β enhances the binding of the astrocyte-derived cytokine interleukin-3 (IL-3) to its receptor, interleukin-3 receptor alpha chain (IL-3Rα), on the microglial membrane, thereby activating signaling mediated by triggering receptor expressed on myeloid cells 2 (TREM2). This leads to enhanced microglial motility and an increased capacity to phagocytose and clear amyloid-β and tau aggregates [45,46]. However, in the later neurotoxic stages of AD, microglial activation promotes the release of proinflammatory cytokines such as interleuking-1 beta (IL-1β) and tumor necrosis factor-alpha (TNF-α), which in turn induce astrocyte transformation into a neurotoxic A1 phenotype [47,48] (Figure 2). Conversely, astrocyte-derived proinflammatory cytokines, including interleukin-15 (IL-15) and interferon-gamma (IFN-γ), have been shown to promote the polarization of microglia toward the M1 phenotype, leading to increased production of IL-1β and TNF-α—cytokines that further stimulate astrocyte activation [48,49]. Moreover, IFN-γ and TNF-α enhance amyloid-β production in astrocytes by upregulating astrocytic BACE1, an enzyme that cleaves APP to generate amyloid-β [49,50]. Therefore, under AD pathology, excessive microglial activation exacerbates the positive feedback loop between microglia and astrocytes, accelerating disease progression through increased release of proinflammatory cytokines and enhanced amyloid-β seeding [40,41,42]. Furthermore, proinflammatory cytokines—including IL-1β, TNF-α, and interleukin-6 (IL-6)—produced by activated microglia and reactive astrocytes have also been shown to accelerate tau protein phosphorylation in AD [51].

## 4. Alterations in Astrocyte Function in Alzheimer’s Disease

**Figure 3:**
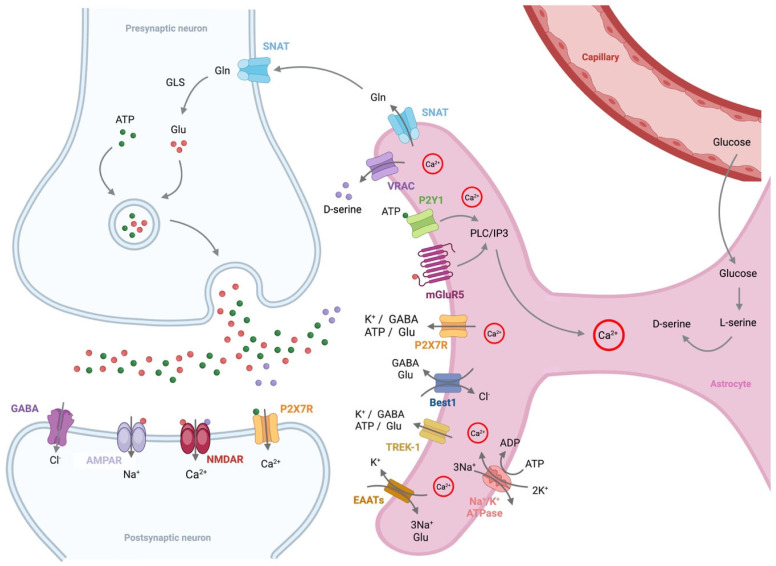
Astrocytes regulate both glutamatergic and GABAergic neuronal functions. Activation of the astrocytic phospholipase C/inositol trisphosphate (PLC/IP3) pathway through mGluR5 and purinergic P2Y1 receptors leads to an increase in astrocytic Ca^2+^ levels. This increase in Ca^2+^ triggers the release of gliotransmitters, including GABA, glutamine (Gln), and D-serine, which are essential for glutamatergic and GABAergic neuronal activity. D-serine, a co-agonist of the NMDA receptor (NMDAR) localized at the postsynaptic surface, is synthesized in astrocytes from glucose supplied by nearby capillaries. In contrast, glutamine, a metabolic precursor of glutamate (Glu), is also produced in astrocytes, and Ca^2+^ activation facilitates its release from astrocytes. The released glutamine is then taken up by neurons through the sodium-coupled neutral amino acid transporter (SNAT), where it is converted into glutamate by the enzyme glutaminase 1 (GLS). The glutamate is then released into the synaptic cleft via presynaptic vesicles to activate AMPA receptor (AMPAR) and NMDAR, both of which are critical for synaptic plasticity, learning, and memory. Additionally, Na^+^/K^+^ ATPases polarize astrocytes by actively pumping 3 Na^+^ ions out and 2 K^+^ ions in, maintaining a negative resting membrane potential. P2X7R, purinergic P2X7 receptor; VRAC, volume-regulated anion channel; Best1, bestrophin-1; TREK-1, TWIK-related K^+^ channel 1; EAATs, excitatory amino acid transporters.

Astrocytes play a crucial role in maintaining synaptic function, neurotransmitter balance, and metabolic support in the brain (Figure 3). In AD, astrocytes undergo significant functional and morphological changes, contributing to disease progression. The Ca^2+^-dependent release of gliotransmitters such as glutamate, D-serine, and adenosine triphosphate (ATP) is elevated in AD, creating an environment that promotes neuronal excitotoxicity [52]. Additionally, Ca^2+^ waves have been shown to propagate across the astroglial network over long distances in AD, suggesting that Ca^2+^ signaling and gliotransmitter release work together to amplify excitotoxic effects [53].

### 4.1. Glutamate

Glutamate is a nonessential amino acid and a major excitatory neurotransmitter that plays a crucial role in maintaining glutamatergic function and neuronal excitability in the brain. This neurotransmitter is primarily synthesized in astrocytes through the Krebs cycle [54]. Therefore, glutamatergic neurons rely on astrocytes to replenish the glutamate pool required for synaptic neurotransmission. Additionally, astrocytes uptake glutamate from the extracellular space through excitatory amino acid transporters (EAATs) for recycling. Since glutamate is excitotoxic, it is converted into glutamine by the enzyme glutamine synthetase before being transported from astrocytes to neurons via sodium-coupled neutral amino acid transporters [54]. In glutamatergic neurons, mitochondrial glutaminase 1, also known as phosphate-activated glutaminase, converts glutamine back into glutamate [54]. The reconverted glutamate is then packaged into synaptic vesicles by vesicular glutamate transporters for subsequent neurotransmission. However, in AD, astrocytic glutamine synthetase levels are reduced, leading to an imbalance in the glutamate/glutamine ratio and exacerbating glutamate-induced excitotoxicity [55]. This disruption in astrocytic glutamate metabolism is thought to be a key factor contributing to AD pathogenesis [55].

### 4.2. D-Serine

N-methyl-D-aspartate (NMDA) receptors play a critical role in synaptic plasticity, learning, and memory. D-serine functions as a co-agonist at the glycine-binding site of NMDA receptors, primarily interacting with the GluN1 subunit. In astrocytes, D-serine is synthesized by serine racemase, which converts L-serine into D-serine. The activation of NMDA receptors requires the binding of both glutamate (to the GluN2 subunit) and D-serine or glycine (to the GluN1 subunit) [56]. Studies have demonstrated that brain D-serine levels play a central role in regulating NMDA receptor activation and its downstream functions [57,58]. However, D-serine levels are significantly higher in postmortem hippocampal and cortical samples from AD patients compared to controls [59]. Similarly, elevated levels of both D-serine and serine racemase have been observed in rodent models of AD [59,60]. Additionally, increased serine racemase expression in reactive astrocytes in AD rats has been associated with heightened NMDA receptor signaling [60]. Studies have also suggested that serum D-serine levels could serve as a potential biomarker for AD progression [61]. Therefore, excessive D-serine levels and NMDA receptor hyperactivation may contribute to the memory deficits and excitotoxicity observed in AD.

### 4.3. ATP

ATP serves not only as a modulator but also as an important neurotransmitter in both the central and peripheral nervous systems [62]. It is a key gliotransmitter released by astrocytes, influencing neuronal activity and synaptic plasticity [63]. ATP mediates fast and slow synaptic potentials through ionotropic P2X receptors and metabotropic P2Y receptors, respectively. In AD, astrocytes undergoing reactive astrogliosis due to amyloid-β accumulation exhibit dysregulated ATP release, leading to abnormal purinergic signaling through P2X and P2Y receptors [64]. Similarly, the application of amyloid-β peptides to hippocampal slices or astrocyte cultures has been shown to induce aberrant ATP release [65]. In addition, astrocytes produce ATP through glycolysis and supply lactate, a byproduct of this process, to neurons via the astrocyte-neuron lactate shuttle to support neuronal metabolism [66]. However, in AD, glucose uptake is reduced due to the decreased expression of astrocytic glucose transporters, and key glycolytic enzymes, such as phosphofructokinase and pyruvate kinase, are downregulated, leading to impaired ATP synthesis [67,68]. Such alterations in ATP production may lead to neuronal energy deficits, synaptic dysfunction, and increased neuroinflammation, potentially accelerating AD progression.

### 4.4. GABA

Gamma-aminobutyric acid (GABA) is the primary inhibitory neurotransmitter in the adult mammalian brain. In addition to neurons, astrocytes also synthesize and release GABA as a gliotransmitter [69]. Astrocyte-derived GABA exerts a tonic inhibitory influence on cerebellar granule neurons and striatal medium spiny neurons via GABA_A_ receptor activation [70], while in the prefrontal cortex, it interacts with GABA_B_ receptors [71]. Under normal conditions, cytosolic GABA concentrations in astrocytes remain low; however, in AD patients and mouse models of AD, GABA levels are abnormally high [72]. Reactive astrocytes surrounding amyloid plaques excessively produce and release GABA through the anion channel bestrophin-1 [73]. This astrocyte-derived GABA inhibits dentate granule neuron activity in the hippocampus, reducing spike probability and contributing to learning and memory impairments [73].

### 4.5. Potassium

The homeostasis of K^+^ is regulated by astrocytes, which play a crucial role in buffering and redistributing K^+^ to maintain ionic balance and ensure proper neuronal function [74]. Neurons release large amounts of K^+^ during firing and synaptic transmission. After neuronal activity, astrocytes take up excess K^+^ from the extracellular space, primarily through inwardly rectifying K^+^ channels (Kir4.1). The K^+^ is then dispersed via gap junctions to areas of lower concentration and subsequently re-released [75]. When extracellular K^+^ levels rise, neurons depolarize, becoming more excitable and vulnerable to excitotoxicity [76]. However, in AD, extracellular K^+^ concentrations are consistently elevated [77], while Kir4.1 expression in astrocytes is reduced [78], leading to impaired K^+^ homeostasis and increased neuronal hyperexcitability. Additionally, a positive correlation has been observed between elevated K^+^ levels and the accumulation of amyloid-β aggregates in AD, suggesting a potential link between disrupted K^+^ regulation and disease progression [79].

### 4.6. Reactive Oxygen

Oxidative stress in astrocytes occurs when there is an imbalance between reactive oxygen species (ROS) production and antioxidant defense mechanisms. ROS, primarily generated in mitochondria, are the main free radicals contributing to oxidative stress and cellular damage [80]. Astrocytic mitochondria play a crucial role in oxidative stress by regulating ROS production, antioxidant defenses, and energy metabolism [81]. Studies have shown that amyloid-β impairs glutamate uptake by astrocytes, leading to excessive glutamate accumulation in the synaptic cleft, overactivation of NMDA receptors, and subsequent oxidative stress via Ca^2+^ influx and ROS production [80,82,83]. Moreover, in AD, astrocytes exhibit dysregulated Ca^2+^ homeostasis, reduced glucose metabolism and mitochondrial ATP synthesis, and increased activity of the ROS-producing enzyme NADPH oxidase [84,85]. These alterations impair astrocytic mitochondrial function, promote excessive ROS production, and exacerbate oxidative damage, ultimately leading to neuronal energy deficits, synaptic dysfunction, and cognitive decline [76].

## 5. Astrocytes in Synaptic Dysfunction in Alzheimer’s Disease

Growing evidence suggests that astrocytes actively modulate synaptic transmission [86,87,88,89], input integration, and neuronal excitability [90]. They also influence spike waveform and axonal conductivity [91,92]. Astrocytes can detect neuronal activity and, depending on action potential firing rates, release gliotransmitters such as adenosine and glutamate [92,93] while also triggering intracellular Ca^2+^ oscillations at different frequencies [94]. However, in AD, reactive astrocytes exhibit upregulated expression of genes associated with synaptic deterioration [25,95,96]. This loss of synaptic integrity may contribute to synaptic dysfunction and cognitive decline [97]. Moreover, amyloid-β has been shown to exacerbate astrocytic glutamate release and drive synaptic loss [98].

### 5.1. Synaptic Plasticity

Synaptic plasticity refers to the ability of synapses to strengthen or weaken over time in response to neural activity [99]. The Hebbian form of synaptic plasticity is the most widely studied type of long-lasting, activity-dependent change in synaptic strength, driven by positive feedback mechanisms [100]. Long-term potentiation (LTP) and long-term depression (LTD) are key examples of Hebbian synaptic plasticity. However, neurons also engage in homeostatic synaptic plasticity, a non-Hebbian form of plasticity, to counterbalance the destabilizing effects of Hebbian plasticity. Homeostatic plasticity employs negative feedback mechanisms to maintain neural network stability by preventing excessive excitation or inhibition [101].

#### 5.1.1. Hebbian Synaptic Plasticity

##### Long-Term Potentiation

Studies have shown that LTP is dependent on astrocytic Ca^2+^ signaling [102]. Choline-induced LTP requires an inositol 1,4,5-triphosphate receptor 2 (IP3R2)-mediated Ca^2+^ increase in astrocytes, and blocking this Ca^2+^ increase prevents LTP induction [103]. This rise in Ca^2+^ stimulates astrocytes to release glutamate, which then binds to metabotropic glutamate receptors (mGlu receptors) on neighboring neurons, leading to their activation [103]. Furthermore, an IP3R2-dependent Ca^2+^ increase in astrocytes is essential for late-phase LTP [104]. In addition, NMDA receptor-dependent LTP in hippocampal CA1 synapses relies on the transient release of D-serine, a physiological co-agonist of NMDA receptor, from local astrocytes [105]. Thus, LTP induction depends on both astrocytic Ca^2+^ signaling and D-serine release [102,105,106]. Additionally, L-lactate released by astrocytes plays a crucial role in hippocampal LTP [107]. Astrocytes also modulate LTP via cannabinoid receptor 1 (CB1 receptor). Like neurons, astrocytes express CB1 receptor, and its activation triggers Ca^2+^-dependent glutamate release, enhancing synaptic transmission at the hippocampal CA3-CA1 synapse [108]. Moreover, CB1 receptor mediates astrocytic D-serine release, which is critical for hippocampal LTP [109]. However, in AD, LTP is altered [110]. Studies show that reactive astrocytes exhibit dysregulated Ca^2+^ signaling, leading to abnormal gliotransmitter release, including glutamate and D-serine, both essential for NMDA receptor activation and LTP [60,111]. Additionally, reduced astrocytic uptake of glutamate in AD due to impaired EAATs results in excitotoxicity, further disrupting synaptic function [112]. Furthermore, astrocytic secretion of BDNF, which supports synaptic strengthening, is downregulated in AD, exacerbating LTP deficits [113].

##### Long-Term Depression

Astrocytes regulate NMDA receptor-dependent LTD by modulating endogenous levels of cannabinoids and D-serine. The neuronal release of endogenous cannabinoids in the hippocampus activates CB1 receptors on astrocytes, which increases extracellular glutamate concentration and activates NMDA receptors on the postsynaptic surface of neighboring neurons. This leads to the internalization of AMPA receptors from the synaptic surface and the induction of LTD [114]. Furthermore, the release of D-serine by astrocytes is also necessary for NMDA receptor-dependent LTD in the hippocampus [115,116]. In contrast to NMDA receptor-mediated LTD, astrocytes are also involved in mGlu receptor-dependent LTD in both the neocortex and hippocampus [117]. Induction of LTD through synaptic stimulation activates astrocytic Ca^2+^ signaling and SNARE-dependent ATP release from astrocytes. The released ATP then activates postsynaptic P2X receptors in neurons, triggering AMPA receptor internalization and synaptic depression [117]. Moreover, cortical high-frequency stimulation increases Ca^2+^ in striatal astrocytes through the activation of mGlu receptors subtype 5, which is necessary for adenosine A1 receptor-mediated LTD [118]. In addition, p38α mitogen-activated protein kinase (MAPK) activity in astrocytes is required for hippocampal LTD [119], while insulin-like growth factor 1 (IGF-1) receptor-mediated activation of astrocytes is essential for cortical LTD [120]. However, LTD is impaired in AD [121,122], and studies have shown that astrocytic Ca^2+^ signaling [111], activation of CB1 receptor-mediated pathways [123], and the release of gliotransmitters such as D-serine, ATP, and glutamate [60,124] all of which are critical for NMDA receptor-dependent and mGlu receptor-dependent LTD—are altered in AD. These astrocytic dysfunctions compromise the mechanisms that support LTD expression [125].

##### Spike-Timing-Dependent Plasticity

Spike timing-dependent plasticity (STDP) is a form of synaptic plasticity in which the timing of action potentials in presynaptic and postsynaptic neurons determines whether synaptic strength is increased or decreased [126]. If the presynaptic neuron fires before the postsynaptic neuron within a critical time window (typically less than 20 ms), synaptic efficacy is enhanced, a process known as spike timing-dependent LTP (t-LTP). Conversely, if the presynaptic neuron fires after the postsynaptic neuron, synaptic strength is weakened, referred to as spike timing-dependent LTD (t-LTD) [126]. Astrocytes modulate both types of STDP and are essential for their expression in the brain [127]. Studies show that the presynaptic form of t-LTD is initially expressed in the hippocampal CA3-CA1 synapse in early development, but as mice mature, this shifts to t-LTP [128]. This developmental transition is driven by the astrocytic release of adenosine and glutamate, which activate presynaptic adenosine A1 receptors and mGlu receptors, respectively [128]. Furthermore, the reversal of presynaptic t-LTP to t-LTD in L4-L2/3 synapses of the adult mouse sensory cortex (S1) upon A1 receptor blockade indicates that adenosine A1 receptors are crucial for t-LTP expression [129]. In contrast, excitatory afferents from the lateral and medial perforant pathways of the entorhinal cortex to dentate gyrus granule cells exhibit two forms of presynaptic t-LTD. While t-LTD at the lateral perforant pathway-granule cell synapse is NMDA receptor-independent, the medial perforant pathway-granule cell synapse requires NMDA receptor activation. However, both forms of t-LTD depend on astrocytic CB1 receptor activity and glutamate release [130]. Additionally, the induction of endogenous cannabinoid-mediated t-LTD in the developing cortex has been shown to depend on CB1 receptor activation and a transient increase in astrocytic Ca^2+^ [131]. Although there is a lack of studies exploring the effect of astrocytes on t-LTP and t-LTD in AD, research has shown that AD patients exhibit impaired STDP following time-locked activation of cortico-cortical connections [132]. Similarly, AD mouse models have demonstrated STDP impairments at Schaffer collateral-CA1 synapses [133] and excitatory synapses of pyramidal cells in neocortical layer 2/3 [134].

#### 5.1.2. Homeostatic Synaptic Plasticity

**Figure 4:**
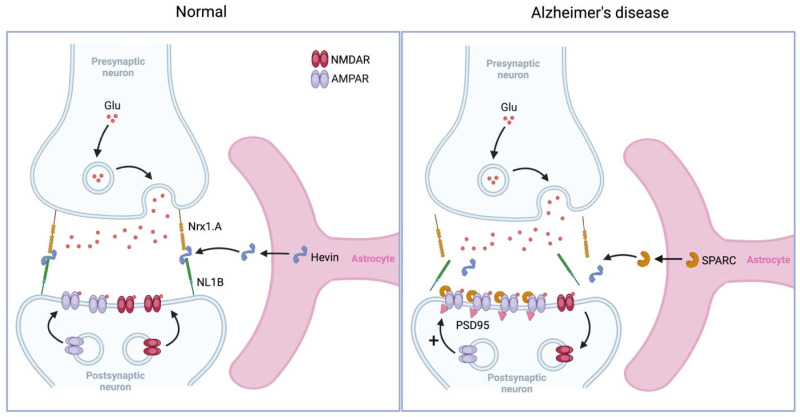
Astrocyte-induced synaptic plasticity. During ocular dominance, astrocyte-secreted SPARC-like protein 1 (Hevin) bridges the cell adhesion molecules Neurexin-1 (Nrx1.A) and Neuroligin-1 (NL1B) to facilitate the recruitment of NMDA receptors (NMDARs) and AMPA receptors (AMPARs) at the postsynaptic surface, promoting Hebbian synaptic plasticity. However, in Alzheimer’s disease, increased astrocytic release of SPARC disrupts NMDAR recruitment by limiting Hevin’s interaction with Nrx1.A and NL1B, while stabilizing AMPARs at the postsynaptic surface, leading to homeostatic synaptic upscaling. Glu, glutamate; PSD95, postsynaptic density 95.

Astrocytes secrete several proteins, including secreted protein acidic and rich in cysteine (SPARC) [135], interleukin-33 (IL-33) [136], and TNF-α [137], which are implicated in homeostatic synaptic scaling. Tetrodotoxin (TTX), a neurotoxin, selectively blocks voltage-gated Na^+^ channels, preventing action potential firing in neurons. As a compensatory mechanism, astrocytes respond to TTX-induced neuronal silencing by releasing specific proteins to restore network excitability. TTX-induced release of TNF-α promotes the insertion of GluA1-containing AMPA receptors at the synaptic surface, facilitating homeostatic synaptic upscaling and enhancing synaptic strength [138,139]. Conversely, TTX-induced release of SPARC and IL-33 facilitates synaptic upscaling by modulating synaptogenesis and upregulating excitatory synaptic components, respectively [136,140]. Additionally, astrocyte-secreted BDNF is also implicated in homeostatic synaptic scaling. BDNF exerts a bidirectional effect on synaptic scaling, where low BDNF levels promote synaptic upscaling by increasing the insertion of AMPA receptors at the synaptic surface, while high BDNF levels facilitate synaptic downscaling by promoting AMPA receptor internalization [141,142,143]. Studies show that amyloid-β enhances the production of TNF-α [144], and the levels of TNF-α [145,146], SPARC [147,148], and IL-33 [149] are elevated in AD. Moreover, amyloid-β triggers homeostatic synaptic overscaling [150], and AD mice exhibit an aberrant upscaling of excitatory neuronal activity in hippocampal circuits [151]. It has been argued that such overactive synaptic scaling may drive disease progression in AD [152]. Therefore, unlike LTP and LTD, which are impaired in AD due to reduced neuronal excitability, synaptic upscaling in AD may result from increased astrocytic secretion of proteins such as SPARC (Figure 4).

### 5.2. Synaptic Remodeling

In addition to regulating synaptic plasticity, astrocytes also influence structural synaptic changes. They play a critical role in the formation of new synapses and the elimination of old synapses [153].

#### 5.2.1. Synaptogenesis

Astrocytes are involved in synaptogenesis, the process of forming new synapses [154]. This process is modulated by astrocyte-secreted molecules, including glypicans, hevin, and thrombospondins [15,155]. Studies have shown that thrombospondin 1 (TSP1) and thrombospondin 2 (TSP2) promote synapse formation in the developing brain by binding to the voltage-dependent Ca^2+^ channel subunit α2δ-1 [154,156]. However, amyloid-β inhibits the release of TSP1 [157], leading to reduced TSP1 levels in the AD brain [158]. Interestingly, increasing TSP1 levels has been shown to prevent synaptic pathology in AD mouse models, highlighting its potential neuroprotective role in the disease [158,159].

#### 5.2.2. Synaptic Pruning

Pruning is the process by which unnecessary or degraded synaptic connections between neurons are eliminated. Astrocytes play a key role in synaptic pruning by engulfing and degrading unwanted synapses. This process is mediated by MER tyrosine kinase (MERTK) and multiple EGF-like domains 10 (MEGF10) receptors, which recognize specific molecular components of degenerating neurons, such as phosphatidylserine, to facilitate astrocytic phagocytosis [160]. MEGF10 knockout mice fail to eliminate functionally impaired excitatory synapses, leading to their excessive accumulation [160]. These knockout mice exhibit impaired long-term synaptic plasticity and deficits in memory formation [161]. However, in Alzheimer’s disease (AD), both MEGF10 and MERTK are downregulated, resulting in impaired elimination of excitatory synapses and dysregulated synaptic transmission [161]. Accordingly, defective astrocytic phagocytosis has been observed in both AD mouse models and human patients [162]. Additionally, astrocytes work in conjunction with microglia to promote synaptic elimination. IL-33, produced by astrocytes, sends signals to microglia to enhance microglial synaptic phagocytosis [163].

## 6. Astrocytes in Memory Deficits in Alzheimer’s Disease

Astrocytes are integral to memory formation and storage through their regulation of synaptic plasticity, neurotransmitter homeostasis, and metabolic support. They release gliotransmitters such as glutamate, D-serine, and ATP, which modulate synaptic strength and LTP, both essential for memory consolidation. Astrocytes also maintain synaptic stability by regulating homeostatic synaptic scaling and clearing excess neurotransmitters. In Alzheimer’s disease, astrocytic dysfunction—including impaired glutamate uptake, disrupted calcium signaling, and altered secretion of trophic factors—contributes to deficits in various types of memory.

### 6.1. Recognition Memory

Recognition memory is the fundamental ability to recognize previously encountered individuals, objects, and events. Studies show that astrocytes contribute to recognition memory [164]. Recognition memory as well as synaptic activity require astroglial glutamine [165], and chemogenetic activation of astrocytes enhances recognition memory [166]. Moreover, inhibition of astrocytic glutamine release through connexin 43 hemichannels impairs recognition memory in mice, and an administration of glutamine reverses this memory deficit glutamine [165]. Glutamine serves as a metabolic substrate for glutamate neurotransmitters and is supplied to neurons by astrocytes. Additionally, the loss of either astrocytic insulin-like growth factor receptor 1 [120] or CB1 receptor [109] in mice results in recognition memory impairments. Activation of the CB1 receptor leads to the release of D-serine from astrocytes. Therefore, exogenous supplementation of D-serine or inhibition of its degradation in CB1 receptor knockout mice results in improved recognition memory [109]. Additionally, reducing endogenous mitochondrial ROS production or inhibiting glycolysis in astrocytes has been shown to result in recognition memory deficits [167,168], underscoring the essential role of brain energy metabolism in this type of memory. The knockout of the p75 neurotrophin receptor, responsible for proBDNF uptake in astrocytes, also alters object recognition memory and hinders late-phase LTP [169]. Recognition memory has been shown to be altered in mouse models of Alzheimer’s disease as well as in patients with Alzheimer’s disease, and this deficit is accompanied by reactive astrogliosis [170,171,172]. In AD, several astrocytic functions are compromised, including glutamine metabolism [173], connexin 43 hemichannels [174], insulin-like growth factor receptor 1 [175], CB1 receptor signaling [123], D-serine production [59], mitochondrial ROS regulation [176], and p75 neurotrophin receptor function [177]. Therefore, impairments in these critical astrocytic processes may contribute to recognition memory deficits in Alzheimer’s disease.

### 6.2. Spatial Memory

Spatial memory is a fundamental cognitive ability that provides the brain with information about the location of objects or events. Astrocytic Ca^2+^ activity in mice increases after exposure to a new environment, and this activity can predict the position of mice in a familiar environment [178]. Studies using two-photon calcium imaging in mice navigating a virtual space have shown that Ca^2+^ signaling encodes spatial information in astrocytes, complementing and synergizing with neuronal encoding [179]. Connexins 30 and 43 form gap junctions that allow direct communication between neighboring astrocytes through Ca^2+^ waves, forming a large network [180]. Double knockout mice lacking these connexins in astrocytes exhibit a complete lack of spatial learning and memory [181]. Similarly, blocking the signal transduction of IL-33, a secreted astrocytic protein involved in hippocampal neurotransmission, causes loss in the formation of spatial memory [182]. Deficits in spatial memory are often an early symptom of Alzheimer’s disease and are considered important factors in assessing functional cognitive disability [183]. Studies in both transgenic mice and humans have shown that spatial memory is altered in AD [184,185]. Moreover, tau accumulation in astrocytes has been shown to induce spatial memory deficits in AD [39]. Furthermore, impairments in glycolysis-derived L-serine production, which serves as a precursor to D-serine—a co-agonist of NMDA receptors required for synaptic function—contribute to spatial memory deficits in AD [186]. In addition, astrocytic Ca^2+^ activity [187], connexin 30 and 43 levels [188], and IL-33 signaling [189] are all impaired in AD, and restoration of IL-33 function has been shown to reverse cognitive deficits [190].

### 6.3. Fear Memory

During fear learning, a subset of astrocytes exhibits elevated Ca^2+^ levels, which persist for the duration of the fear memory and diminish upon extinction of the learned behavior [191]. This astrocytic responsiveness requires the activation of the α7 subunit of nicotinic acetylcholine receptors, and the knockout of this receptor subunit in astrocytes impairs fear memory [191]. Activation of astrocytes via channelrhodopsin-2 (ChR2), a light-sensitive ion channel that facilitates cation influx and subsequent astrocytic activation, induces extracellular Ca^2+^ influx in astrocytes. This leads to the release of adenosine and activation of adenosine A1 receptors, resulting in impaired fear memory [192]. Astrocytic Gq protein-mediated Ca^2+^ increases in the amygdala impair fear memory through activation of adenosine A1 receptors [193], and knockout of astrocytic muscarinic acetylcholine M1 receptor, a Gq protein-coupled receptor, causes impairment in contextual fear memory in mice [194]. Furthermore, blocking or deleting astrocytic IP3 receptor 2 (IP3R2), which mediates Gq protein-dependent release of intracellular Ca^2+^ from the endoplasmic reticulum, also impairs spatial memory [104,195]. Additionally, a decrease in astrocytic L-lactate production—a glycolysis byproduct supplied to neurons via the astrocyte-neuron lactate shuttle to support neuronal metabolism—and downregulation of monocarboxylate transporters responsible for lactate transfer from astrocytes to neurons result in loss of fear memory [107]. Studies have shown that fear memory is impaired in AD [196,197], and in AD, several factors implicated in fear memory are compromised, including astrocytic L-lactate production and its transportation via monocarboxylate transporters [198,199], adenosine levels [200], α7 subunit of nicotinic acetylcholine receptors [201], adenosine A1 receptors [202], muscarinic acetylcholine M1 receptors [203], and IP3R2 [204]. Moreover, exogenous amyloid-β deregulates Ca^2+^ homeostasis in astrocytes [205,206], and astrocytic Ca^2+^ signaling is altered in AD mice [111]. Additionally, the retraction of perisynaptic astrocytic processes from hippocampal synapses is essential for the consolidation of fear memory; however, this retraction is absent in AD mice [207].

### 6.4. Working Memory

Working memory is a cognitive process responsible for temporarily holding and manipulating information necessary for complex tasks such as learning, reasoning, and comprehension, and it enables to retain and process information over short periods, typically lasting seconds. Astrocytes contribute to working memory by modulating their intracellular Ca^2+^ levels through Gq protein-coupled receptor signaling pathways. Adjustments in Ca^2+^ levels can enhance or impair working memory capabilities. Both optogenetic and chemogenetic activation of Gq signaling in hippocampal astrocytes have been shown to enhance working memory [71,208]. Conversely, inhibition of Gq signaling or deletion of IP3R2, which mediates Gq signaling-induced Ca^2+^ release from intracellular stores, impairs working memory [195,209]. Additionally, mice with a genetic deletion of astrocytic Gq protein-coupled GABA_B_ receptors exhibit working memory deficits [71], underscoring the pivotal role of astrocytic Gq signaling in cognitive processes. Beyond Gq signaling, other astrocytic mechanisms influence working memory. For instance, deletion of astrocytic Gs protein-coupled adenosine A2A receptors [210], astrocyte-specific knockout of IGF-1 receptor [211], blocking of astrocytic gap junction connexin 43 hemichannels [212], and reduced activity of astrocytic mitochondrial superoxide dismutase 2 (SOD2) [213] have all been associated with working memory impairments. Working memory is impaired in mouse and monkey models of AD, as well as in patients with AD [204,214,215,216], and several astrocytic components that facilitate working memory are altered, including IP3R2, GABA_B_ [217,218], adenosine A2A receptors [219], IGF-1 receptors [220,221], connexin 43 hemichannels [222], and mitochondrial SOD2 [223]. Moreover, overexpression of SOD2 prevents memory deficits in AD mice [224], whereas a reduction in SOD2 accelerates the onset of behavioral deficits [225].

## 7. Astrocytes in the Treatment of Alzheimer’s Disease

Like other neurodegenerative diseases Alzheimer’s disease affects astrocytic homeostatic and neuroprotective functions, and the recuperation of these astrocytic functions has been viewed as a viable therapeutic strategy [226]. Although the development of drugs targeting directly at astrocytes remains in nascent stage, studies have shown that oral administration of L-serine, a precursor of D-serine produced by glycolysis in the astrocytes, prevents synaptic and memory impairments in a mouse model of Alzheimer’s disease [186]. Furthermore, using a vector-based gene therapy designed to target astrocytes, a deletion of astrocytic signal transducer and activator of transcription 3 (STAT3), which mediates reactive astrogliosis in Alzheimer’s diseases, decreases amyloid-β levels and plaque burden but also ameliorates memory deficits in AD mice [227]. Additionally, drugs that are initially used for other treatments have also shown promising results. A treatment with neuroprotective drug Riluzole and antibiotic β-lactam augment the expression of astrocytic glutamate transporters, which are critical for maintaining synaptic function and preventing excitotoxicity by efficient glutamate neurotransmitter recycling [228,229], and they are downregulated in Alzheimer’s disease [230].

## 8. Concluding Remarks

**Figure 5:**
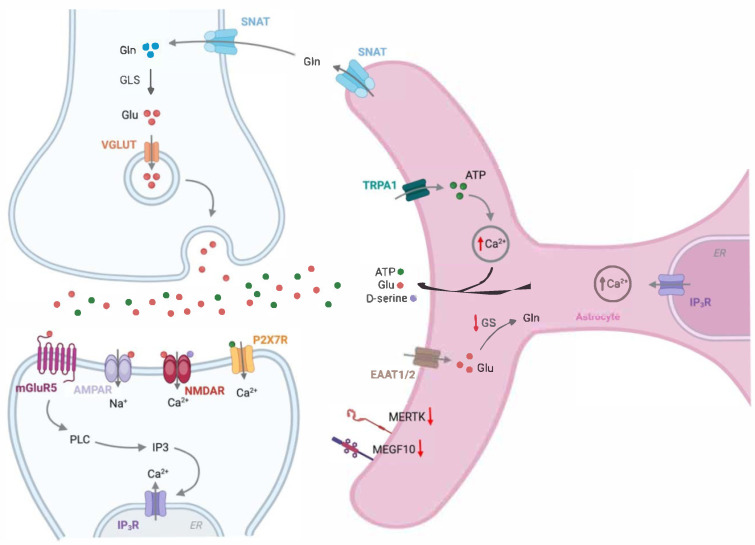
Alterations in astrocyte-neuron communication in Alzheimer’s disease. Upregulation of the astrocytic purinergic P2Y1 receptor, nicotinic acetylcholine α7 receptor, and/or mGlu receptor 5 in AD augments spontaneous astrocytic calcium signals. Elevated astrocytic intracellular Ca^2+^ levels promote the enhanced release of gliotransmitters, such as glutamate, ATP, and GABA. Moreover, astrocytes in AD express lower levels of the glutamate transporters (EAAT1/2), responsible for the uptake of excess glutamate (Glu), contributing to its accumulation in the synaptic cleft. The increase in Glu, D-serine, and ATP in the synaptic cleft overstimulates excitatory glutamate receptors, leading to potential neuronal death. Additionally, the expression of MER tyrosine kinase (MERTK) and multiple EGF-like domains 10 (MEGF10), both essential for synaptic remodeling, is reduced in AD. Thus, astrocyte dysfunction in AD causes impairments in basal synaptic transmission and the induction of synaptic plasticity, leading to deficits in learning and memory. Gln, glutamine; GLS, glutaminase 1; IP3R, inositol trisphosphate receptor; GS, glutamine synthetase; SNAT, sodium-coupled neutral amino acid transporter; EAAT1/2, excitatory amino acid transporter 1/2; TRPA1, transient receptor potential ankyrin 1.

Recent studies suggest that astrocytes are not merely passive support cells but active regulators of spine formation, elimination, and synaptic transmission. Their ability to secrete synaptogenic factors, regulate neurotransmitter availability, and shape neuronal connectivity renders them indispensable for synaptic plasticity and memory functions. Astrocytes play a crucial role in modulating Hebbian forms of synaptic plasticity—such as LTP, LTD, and STDP—as well as non-Hebbian homeostatic synaptic plasticity, which are key mechanisms facilitating learning and memory formation. In AD, astrocytes become reactive and adopt an A1 neurotoxic phenotype, leading to the overproduction of pro-inflammatory cytokines and reactive oxygen species. This phenotypic shift disrupts the metabolism and release of D-serine—a co-agonist of NMDA receptors—and glutamine, which glutamatergic neurons convert into glutamate, the primary excitatory neurotransmitter in the brain (Figure 5). Such imbalances in glutamate and D-serine availability can result in dysfunctions in excitatory neurotransmission, trafficking of synaptic AMPA receptors, synaptic connectivity, and strength, ultimately leading to memory deficits. Additionally, amyloid-β accumulation between neurons and astrocytes can disrupt their interactions, which are critical for neuronal functions involved in synaptic plasticity. Studies in the cerebral cortex and hippocampus have demonstrated that synaptic dysfunction and impaired synaptic plasticity are prominent features of AD [231]. Despite advancements in understanding the role of astrocytes in memory processing and synaptic plasticity, research specifically addressing their involvement in these processes in AD remains limited. Therefore, continued investigation into the role of astrocytes in synaptic and memory functions in AD is essential for understanding how they modulate various forms of synaptic plasticity and contribute to different types of memory formation in the brain. Such research is also crucial for identifying therapeutic targets for the treatment of memory deficits and AD.

## Data Availability

Not applicable.
